# A CRISPR/LbCas12a-based method for detection of bacterial fruit blotch pathogens in watermelon

**DOI:** 10.1128/spectrum.03846-23

**Published:** 2024-02-01

**Authors:** Zelu Wang, Wenhui Cheng, Zhiyu Dong, Xiamei Yao, Xu Deng, Chun Ou

**Affiliations:** 1 Engineering Technology Research Center of Anti-aging Chinese Herbal Medicine, School of Biology and Food Engineering, Fuyang Normal University, Fuyang, Anhui, China; 2 School of Architecture and Urban Planning, Anhui Jianzhu University, Hefei, Anhui, China; 3 Southern Subtropicals Grops Research Institute, Zhanjiang, Guangdong, China; Dominican University New York, Orangeburg, New York, USA

**Keywords:** CRISPR/LbCas12a, *Acidovorax citrulli*, free-amplification, visualization, specificity, sensitivity

## Abstract

*Acidovorax citrulli* is the main pathogen causing bacterial fruit blotch, which seriously threatens the global watermelon industry. At present, rapid, sensitive, and low-cost detection methods are urgently needed. The established CRISPR/LbCas12a visual detection method can specifically detect *A. citrulli* and does not cross-react with other pathogenic bacteria such as *Erwinia tracheiphila*, *Pseudomonas syringae*, and *Xanthomonas campestris*. The sensitivity of this method for genomic DNA detection is as low as 0.7 copies/μL, which is higher than conventional PCR and real-time PCR. In addition, this method only takes 2.5 h from DNA extraction to quantitative detection and does not require complex operation and sample treatment. Additionally, the technique was applied to test real watermelon seed samples for *A. citrulli*, and the results were contrasted with those of real-time fluorescence quantitative PCR and conventional PCR. The high sensitivity and specificity have broad application prospects in the rapid detection of bacterial fruit blotch bacterial pathogens of watermelon.

**IMPORTANCE:**

Bacterial fruit blotch, *Acidovorax citrulli*, is an important seed-borne bacterial disease of watermelon, melon, and other cucurbits. The lack of rapid, sensitive, and reliable pathogen detection methods has hampered research on fruit spot disease prevention and control. Here, we demonstrate the CRISPR/Cas12a system to analyze aspects of the specificity and sensitivity of *A. citrulli* and to test actual watermelon seed samples. The results showed that the CRISPR/Cas12a-based free-amplification method for detecting bacterial fruit blotch pathogens of watermelons was specific for *A. citrulli* target genes and 100-fold more sensitive than conventional PCR with quantitative real-time PCR. This method provides a new technical tool for the detection of *A. citrulli*.

## INTRODUCTION

Bacterial fruit blotch is caused by the Gram-negative bacterium *Acidovorax citrulli*, which is mainly transmitted through contaminated seeds, infected plants, and alternative hosts (1), and can cause devastating diseases in Cucurbitaceae plants (2, 3), seriously endangering global watermelon production (4). So far, there are few reliable methods to control fruit blotch (5). Rapid, sensitive, and low-cost pathogen detection is an important means of disease diagnosis and management strategies, which can minimize the damage caused by *A. citrulli* to watermelon and other cucurbit crops.

At present, the detection methods of plant pathogens include microscopy, immunology, serology, molecular biology, electrochemical sensor technology, immune colloidal gold technology, and so on. As one of the most common molecular biology methods, nucleic acid detection mainly includes nucleic acid molecular hybridization, polymerase chain reaction (PCR), isothermal amplification, gene sequencing, and biochip technology (6–8). With the establishment of new detection methods, nucleic acid detection is developing in the direction of single molecule sensitivity, single base specificity, rapid, and simple. With conventional PCR or quantitative real-time PCR (qPCR), the molecular technology of qPCR is cumbersome and the detection cost is high. It is difficult for the instrument to carry out a wide range of on-site detection. Immunological methods are fast and easy to operate, but the sensitivity is low (9). Simpler and more sensitive diagnostic analysis methods based on isothermal amplification technology have become the mainstream of rapid detection of pathogens. Although isothermal amplification technology helps to reduce some limitations, they need to design relatively complex primers and may also produce false positive results or non-specific amplification (10). Therefore, it is hoped to achieve rapid and sensitive detection of seed-borne pathogens through more direct and simple methods.

Clustered regularly interspaced short palindromic repeats-associated proteins (CRISPR/Cas) system, as a novel gene editing technology, has led to revolutionary progress in genome editing and genetic engineering and is widely welcomed as a rapid, accurate, and robust nucleic acid targeting platform (11). The application of CRISPR/Cas system in the field of biotechnology has exploded, and it is gradually suitable for nucleic acid detection (12). At the same time, compared with qPCR detection methods that take hours, CRISPR/Cas-based SHERLOCK, DETECTR, and FELUDA rapid diagnostic tools have higher accuracy, specificity, and sensitivity and are becoming an inevitable tool for the rapid detection of pathogens (13–15). According to the literature, the Cas12a system is suitable for pathogen detection (16–18). Bai et al. used the nicking enzyme-assisted amplification combined with the CRISPR/Cas12a system to specifically recognize the amplified product and perform a cleavage reaction (19). Salmonella in artificially infected egg fluid can be detected by visual fluorescence observation. Peng et al. used CRISPR/Cas12a for detection with high sensitivity and specificity after target amplification of *Staphylococcus aureus* using PCR (20). Jiao et al. established a detection method for the preference of PAM sites by recombinase polymerase amplification (RPA)-LbCas12a-5M (16), which was used for visual detection of quarantine pathogens *Erwinia amylovora* and *A. citrulli*.

**Fig 1:**
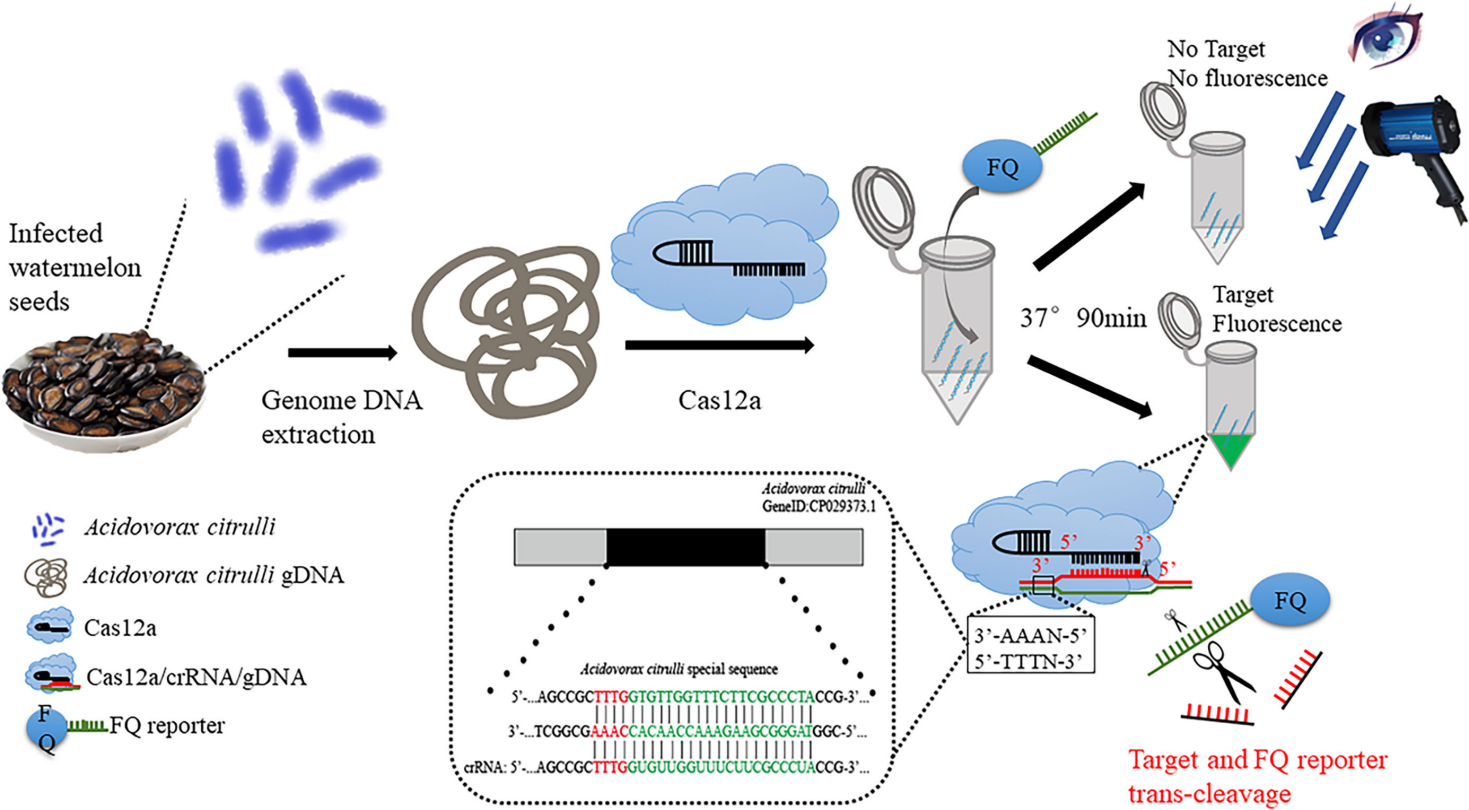
The workflow of CRISPR/LbCas12a detection of *A. citrulli* in this study. Fluorescence changes of watermelon seeds with bacterial fruit blotch extracted from bacterial genomic DNA and detected using the method CRISPR/LbCas12a free-amplification system established in this study, as well as a schematic diagram of fluorescence signal generation of the CRISPR/LbCas12a-crRNA-gDNA ternary complex, as observed by the naked eye under UV light irradiation.

At present, more research on Cas12 is to achieve super-sensitivity detection after amplification, but there are few studies on target sequence detection without amplification. We used the kit method to prepare bacterial genomic DNA to quickly detect *A. citrulli*. In this study, the specificity and sensitivity of CRISPR/Cas12a system for detecting *A. citrulli* were analyzed, and the actual watermelon seed samples were detected. The results were consistent with those of conventional PCR and qPCR. This study is helpful to better understand the construction of the detection method of melon fruit blotch pathogen based on CRISPR/Cas12a system and provides a reference for the study of new detection methods of bacterial fruit blotch pathogen. The flow scheme of CRISPR/LbCas12a detection in watermelon seed samples in this study is shown in (Fig. 1). This ribonuclease cleavage functions through the target gene. The presence of the target gene DNA activates the catalytic domain of the Cas12 protein through specific crRNA hybridization, thereby cutting the nearby single-stranded DNA (ssDNA) reporter gene (21). Using the random reporter gene ssDNA and terminal labeled FAM (F) fluorophore and BHQ1 (Q) quencher, the fluorescence signal released by the CRISPR/LbCas12/crRNA complex binding to the target DNA was monitored by restoring the fluorescence of the FAM fluorophore. The results can be observed by the naked eye with a handheld UV lamp.

## MATERIALS AND METHODS

### Testing material

The standard strain of *A. citrulli* (ACCC 05732) was purchased from China Agricultural Microorganism Culture Collection and Management Center. *Erwinia tracheiphila* (NCPPB 2452), *Pseudomonas syringae* (NBRC 14078), *Xanthomonas campestris* (ATCC 11672), and watermelon seed samples were provided by Ningbo Weimeng Seed Industry Co., Ltd. (Ningbo, China). Oligonucleotide sequences, double distilled water, diethyl pyrocarbonate, deoxyribonucleoside triphosphate (dNTP), rTaqDNA polymerase, 4S Gelred nucleic acid dye, DNA marker (100–1,000 bp), and agarose were purchased from Shanghai Kehua Bioengineering Co., Ltd. (Shanghai, China). CRISPR/LbCas12a (LbCpf1), crRNA, 10 × LbCas12a buffer [150 nM NaCl; MgCl_2_, 100 mM Tris-HCl (pH 9.0), 0.5% Tween-20, 10 mM DTT], and FQ-reporter (5′-FAM-TTATT-BHQ1-3') double-end labeled probes were purchased from Guangzhou Bolaisi Biotechnology Co., Ltd. (Guangdong, China). The bacterial genomic DNA extraction kit was purchased from Tiangen Biochemical Technology Co., Ltd. (Beijing, China). Fluorescence quantitative kit iTaq Universal SYBR Green Supermix was purchased from Bole Life Medical Products Co., Ltd. (Shanghai, China). All reagents were analytically pure and could be used without further purification.

### Bacterial genomic DNA extraction and PCR amplification system

*A. citrulli* was grown in nutrient agar (NA) medium [peptone, beef extract, sodium chloride, agar, distilled water (pH 7.0)] at 28°C for 48 h under sterile conditions. Single colonies were selected and placed in a test tube containing liquid NA medium (without agar). The test tube was placed on a shaker at 150 rpm for 48 h, and 1 mL of the bacterial solution was taken. After centrifugation at 12,000 rpm for 10 min, the bacteria were obtained for molecular detection, according to the Tiangen bacterial genomic DNA extraction kit instructions. The concentration and copy number of Tris-EDTA buffer-eluted DNA were measured with a Thermo NanoDrop 2000 ultramicro spectrophotometer. The copy number calculation formula is as follows: DNA copy number = (6.02 × 10^23^) × (M × 10^−9^) / (*n* × 660) (22) (M is the concentration measured by spectrophotometer, and *n* is the genome base logarithms). The conserved region of 16S-23S internal transcribed spacer (ITS) of *A. citrulli* (GeneID: CP029373.1 1557610–1557855) was used as the target gene. The conventional PCR amplification system was 25 µL, 2.5 µL of 10 × PCR buffer, 2 µL of 2.5 mM dNTP, 0.15 µL of 5 U/µL rTaqDNA polymerase, 0.5 µL of 10 µmol/µL upstream and downstream primers, primers sequence as shown in Table 1 , and 1 μL of template DNA (each concentration gradient dilution). The final volume was supplemented to 25 µL with ddH_2_O. Reaction procedure is as follows: 95°C for 5 min, 95°C denaturation for 30 s, 53°C annealing for 30 s, 72°C extension for 30 s, 35 cycles, and 72°C final extension for 5 min. The PCR amplification products were subjected to 1% agarose gel electrophoresis, and the results were observed and imaged by Bole Gel Doc EZ gel imager. The qPCR amplification system was 25 µL, 12.5 µL of iTaq Universal SYBR Green Supermix, and 0.5 µL of 10 µM forward and reverse primers. Primers were consistent with conventional PCR primers (sequence shown in Table 1) and 1 µL template DNA (diluted with each concentration gradient), and the final volume was supplemented with ddH_2_O to 25 µL. Reaction procedure is as follows: 95°C for 10 min, denaturation at 94°C for 15 s, annealing at 53°C for 1 min, and 35 cycles.

**TABLE 1:** Primer, crRNA, and probe reporter sequences

Name	Sequence (5′−3′)	Resource
SEQID4^m^	GTCATTACTGAATTTCAACA	(23)
SEQID5	AGCGTATTGGTTGGTGGAGG	(23)
crRNA	UAAUUUCUACUAAGUGUAGAUGUGUUGGUUUCUUCGCCCUA	This work
FQ	FAM-TTATT-BHQ1	This work

### Synthesis of crRNA and fluorescent reporter FQ

According to the 16S-23S ITS region, we used Primer Premier 5 to design a species-specific crRNA targeting sequence. In the target gene-specific fragment, BLAST (National Center for Biotechnology Information) analysis confirmed the conservation of the sequence within the species and the specificity between species. The crRNA targeting sequence must exist in the amplification product and cannot overlap with the primer to avoid false positive results in negative samples (23, 24). crRNA recognizes the 20-nt sequence adjacent to the “TTTN” (N is any base) site, and crRNA and ssDNA fluorescent reporter modified double-ended FAM-BHQ1 probes were designed (the sequence is shown in Table 1).

### Cas12a detection reaction

The detection system was 25 µL, and 0.5 µL of 5 µM LbCas12a, 2.5 µL of 10 µM crRNA, and 2.5 µL of 10 × LbCas12a buffer were mixed and incubated at 37°C for 15 min to form LbCas12a/crRNA binary complex. After that, 2 µL of template DNA and 2.5 µL of 10 µM FQ-reporter were added under low light to form the target ternary complex, and the volume was replenished to 25 µL with diethyl pyrocarbonate water.

### Specific detection

The obtained *E. tracheiphila*, *P. syringae*, and *X. campestris* were cultured in broth (LB) medium (peptone, yeast powder, sodium chloride, agar, and distilled water, pH 7.0), growth conditions, and DNA extraction (the same as the above bacterial culture and genomic extraction above), and the DNA template concentration of different bacteria was adjusted to be consistent. CRISPR/LbCas12a system was used to detect *A. citrulli*, *E. tracheiphila*, *P. syringae*, *X. campestris* and other common pathogens of cucurbitaceous plants to verify the specificity of crRNA.

### Sensitivity detection

The genomic gDNA was extracted by the kit method as template DNA. The initial concentration of gDNA was 7 × 10^6^ copies/μL and was diluted with ddH_2_O to 7 × 10^5^ copies/μL, 7 × 10^4^ copies/μL, 7 × 10^3^ copies/μL, 7 × 10^2^ copies/μL, 7 × 10^1^ copies/μL, 7 × 10^o^ copies/μL, 7 × 10^−1^ copies/μL, 7 × 10^−2^ copies/μL, and other different concentrations of DNA templates. The extracted genomic DNA was converted to copy number and was detected by the established method to evaluate the detection limit. At the same time, SEQID4^m^/5 primers (Table 1) were used to detect the template by conventional PCR and qPCR, and the sensitivity of the three methods was compared.

### Detection of actual watermelon seed samples

Nine watermelon seeds suspected to be infected with *A. citrulli* were randomly selected and detected by the detection method established in this study, conventional PCR, and qPCR. The *A. citrulli* genomic DNA was used as a positive control, and ddH_2_O was used as a negative control. The results of the two methods were compared.

## RESULTS

### Specificity analysis of CRISPR/LbCas12a system

**Fig 2:**
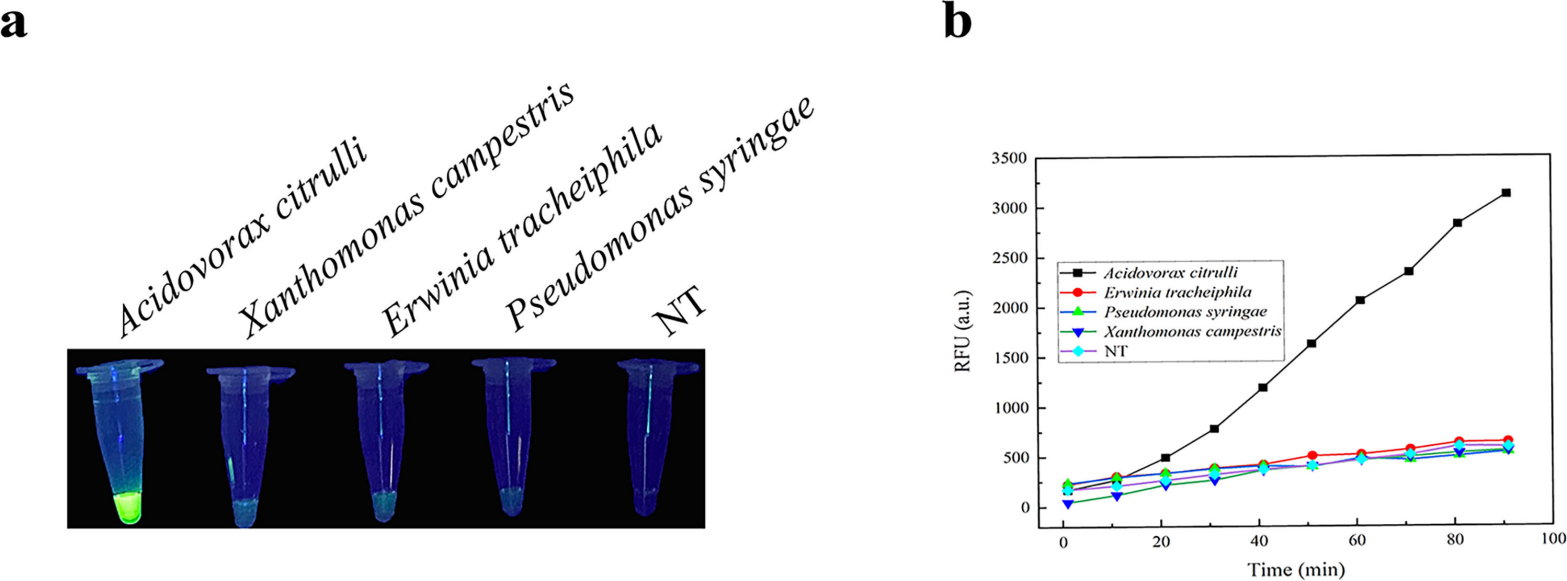
Analysis of CRISPR/LbCas12a/crRNA for specific detection of *A. citrulli*. (a) Visualization of fluorescence signals of *A. citrulli*, *E. tracheiphila*, *P. syringae*, and *X. campestris* under handheld UV light irradiation. (b) Quantification of CRISPR/LbCas12a system by enzyme marker for detection of *A. citrulli*, *E. tracheiphila*, *P. syringae*, and *X. campestris* fluorescence signals.

The CRISPR/LbCas12a system uses reagents and equipment that are readily available in most routine laboratories, so it can detect bacterial samples. The CRISPR/LbCas12a system is used to detect common cucurbit pathogens: *E. tracheiphila*, *P. syringae*, and *X. campestris* genomic DNA, aligning concentration regulation, as non-target genes with *A. citrulli* (25). The specificity of the CRISPR/LbCas12a system for *A. citrulli* was tested by *E. tracheiphila*, *P. syringae*, and *X. campestris* and was verified. The results are shown in (Fig. 2a). Under the irradiation of a handheld UV lamp, it was observed that the CRISPR/LbCas12a system could produce significant fluorescence for *A. citrulli* gDNA, while no fluorescence was produced for the other three non-target genes. The quantitative results are shown in (Fig. 2b). The fluorescence intensity of the CRISPR/LbCas12a system for *A. citrulli* increased with increasing reaction time of the system, while no fluorescence signal was produced for the other three tested strains. The results indicate that the CRISPR/LbCas12a system is highly specific for the detection of *A. citrulli*.

### Sensitivity analysis of CRISPR/LbCas12a system

**Fig 3:**
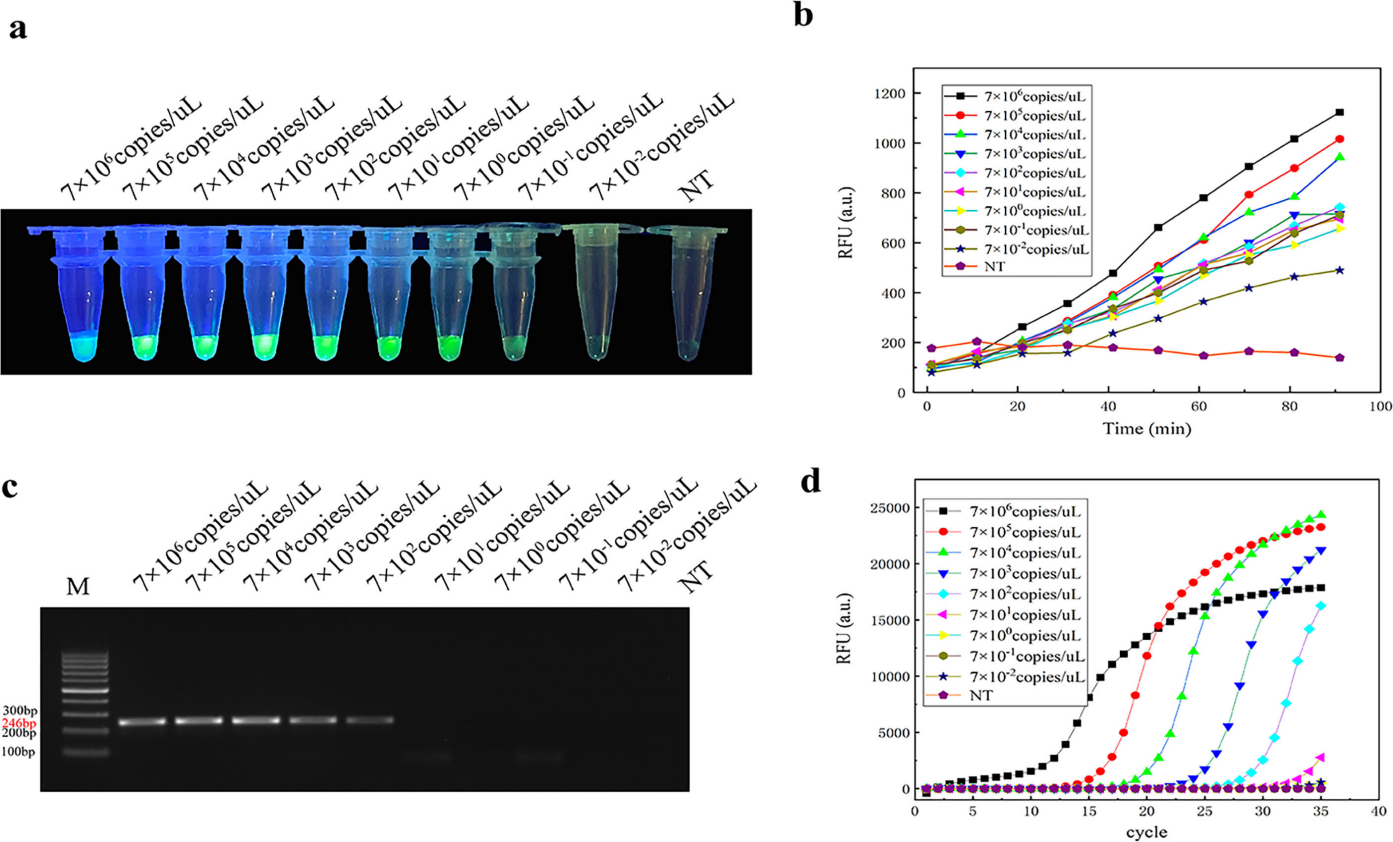
Sensitivity of CRISPR/LbCas12a/gDNA free-amplification comparative analysis with PCR sensitivity. (a) Free-amplification visualization results for each concentration of gDNA-CRISPR/LbCas12a under a handheld UV lamp, and the fluorescence diminished with decreasing concentration, with a significant reduction at 7 × 10^−1^ copies. (b) Quantitative analysis results for different concentrations of gDNA-CRISPR/LbCas12a with a detection limit up to 0.7 copies. (c) Analysis of the results of conventional PCR gel electrophoresis at different concentrations; M: DL1000 DNA maker, conventional PCR gradient dilution, initial concentration of 7 × 10^6^ copies, and the detection limit of 7 × 10^2^ copies. (d) Sensitivity analysis of qPCR at different concentrations, the detection limit of qPCR in the figure is 7 × 10^2^ copies.

The different detection methods were validated against the method established in this study. The results showed that the visual observation was significantly diminished at 7 × 10^−1^ copies under the handheld UV lamp (Fig. 3a). A concentration dependence was shown when 7 × 10^5^–7 × 10^−2^ copies were made. The fluorescence signal decreased linearly with decreasing concentration. The quantitative results were comparable to the fluorescence intensity of the negative control at 7 × 10^−1^ copies, so the detection limit reached 7 copies. The visual results were in general agreement with the quantitative analysis (Fig. 3b). Therefore, the detection limit of the assay established in this study was 0.7 copies.

### Comparison of the sensitivity of conventional PCR and qPCR with the method established in this study

Compared with conventional PCR and qPCR, the fluorescence curves of each concentration gradient were compared by qPCR with a Cq value ≤35 as the boundary. The detection limit was 7 × 10^2^ copies (Fig. 3c). Imaging of conventional PCR was performed by gel electrophoresis. A clear band appeared at 7 × 10^6^–7 × 10^3^ copies, while the band at 7 × 10^2^ copies was not bright, indicating that the detection limit of conventional PCR was 7 × 10^2^ copies. qPCR results (Fig. 3d) showed that at 7 × 10^6^ copies, a peak started to appear in 10 cycles of qPCR due to the high concentration, and the peak time was prolonged as the concentration decreased. At 7 × 10^1^ copies, the fluorescence intensity was below the instrument baseline and reached the threshold, which is consistent with the detection limit of conventional PCR. The qPCR sensitivity Ct values are shown in Table 2 below.

**TABLE 2:** qPCR sensitivity Ct value

Concentration gradient (copies/μL)	Ct values
7 × 10^6^	13.06
7 × 10^5^	17.62
7 × 10^4^	21.68
7 × 10^3^	26.42
7 × 10^2^	30.82

The results show that the CRISPR/LbCas12a free-amplification system established in this study is more sensitive than conventional PCR and qPCR, which can not only quickly detect the target sequence of low-concentration pathogenic bacteria, but also has low cost and is more widely used in the field detection combined with visual inspection.

### CRISPR/LbCas12a system for detection of pathogenic bacteria in watermelon seeds

**Fig 4:**
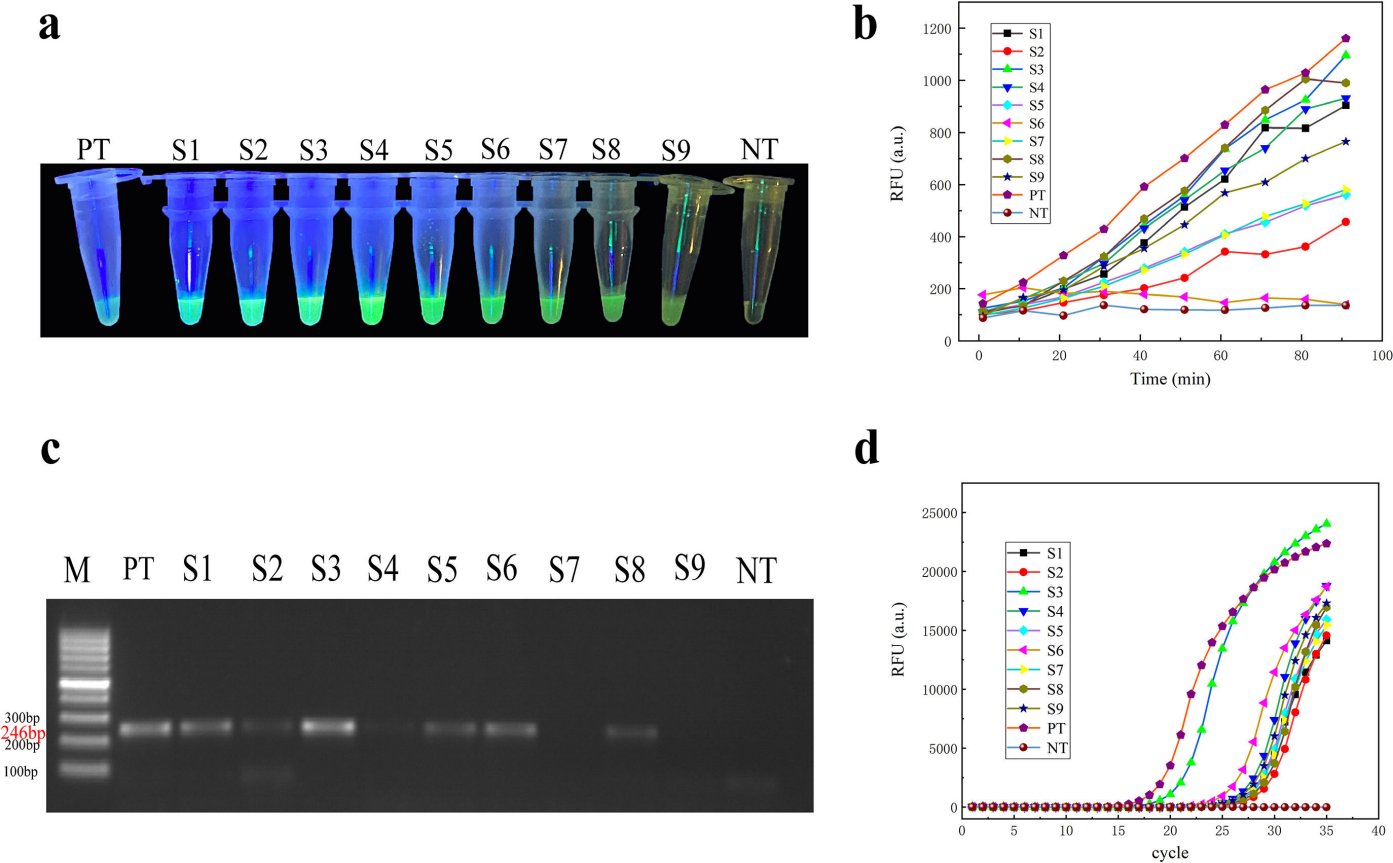
Actual sample detection and analysis of seeds with *A. citrulli*. (a) Watermelon seeds’ CRISPR/LbCas12a handheld UV light visual observation results. (b) Quantitative detection and analysis by enzyme marker. (c) Conventional PCR detection and analysis, and NT as negative control. (d) qPCR detection and analysis.

Nine watermelon seed samples suspected to be infected by *A. citrulli* were collected from Ningbo and other places, and four detection methods were compared (Fig. 4). From the results, handheld UV light visual detection showed that the naked eye was to observe the light (Fig. 4a) S3, S4 fluorescence is the brightest, the other several kinds of seed fluorescence are present, and the naked eye cannot distinguish; quantitative detection (Fig. 4b) showed that the fluorescence intensity of S3 and the positive control was higher, indicating that the amount of *A. citrulli* in S3 seeds was higher, and the fluorescence intensity of other seeds was higher than that of the negative control.

### Comparison of conventional PCR and qPCR with the method established in this study for the detection of actual watermelon seeds

Conventional PCR (Fig. 4c) showed that only S3 had a clear band, S4 had a weak band, and S7 and S9 were not obvious, indicating that the concentration of seed samples with *A. citrulli* target genes was too low for conventional PCR detection to reach its limit. qPCR results (Fig. 4d) showed that the fluorescence intensity of S3 was higher than that of the positive control, and the fluorescence signals of the other samples were similar. Nine watermelon seeds with different amounts of cultured bacteria were detected, and the threshold was reached by conventional PCR, which was not detectable for S7 and S9 seeds. qPCR detected eight seeds peaking at around 30 cycle counts. Sample qPCR Ct values are shown in Table 3 below. Unlike PCR, the method established in this study was able to detect seeds with lower target genes, and the generation of fluorescent signals could be observed under a handheld UV lamp, allowing the detection of bacterial fruit blotch in watermelon seeds without relying on large instruments and also without complex expertise techniques.

**TABLE 3:** Actual sample qPCR Ct values

Test samples	Ct values
S1	29.72
S2	30.64
S3	22.14
S4	28.90
S5	29.64
S6	27.42
S7	29.85
S8	30.17
S9	29.29

We compared previously reported Cas12a assays for pathogenic bacteria (Table 4). Unlike conventional PCR assays, amplification is not required. In addition, our assay is simple, rapid, and more widely applied highly achievable for target gene detection without relying on large instruments. This PCR, qPCR, and the method established in this study were used to detect and analyze watermelon seed samples with consistent results. It could also be detected at low concentrations in the target seeds assay, being more sensitive than PCR, which again proved the feasibility of the method established in this study.

**TABLE 4:** Comparison of Cas enzyme system assays

Name	Cas enzyme	Target	Organism	Amplification	Sensitivity	Time for detection	Reference
HOLMES	Cas12a	DNA, RNA	JEV, pseudorabies virus (PRV)	PCR	10 aM	1 h	(25)
HOMESV2	Cas12b	DNA, RNA	JEV, pseudorabies virus (PRV), human SNPs	LAMP	~10 aM	1 h	(26)
Cas12a fluorescent based point of care system	Cas12a	DNA	ASFV	No	1 pM	2 h	(27)
E-CRISPR	Cas12a	DNA	HPV16	No	50 pM	/	(28)
CRISPR/Cas12a-mediated dual-mode electrochemical biosensor	Cas12a	DNA	Modified soybean	No	0.3 ~ 3 fM	<1 h	(29)
Amplification-free detection of SARS-CoV-2 with CRISPR-Cas13a and mobile phone microscopy	Cas13a	RNA	SARS-CoV-2	No	~100 copies/μL	<1 h	(30)
Our study	Cas12a	DNA	*Acidovorax citrulli*	No	0.7 copies/μL	1.5 h	This work

## DISCUSSION

Bacterial fruit blotch disease of watermelons can infect cucurbits and cause devastating damage; it also is a listed quarantine pest in China. To increase the rate of disease interception and expedite customs clearance, rapid and precise detection technology is critical. CRISPR/Cas12a nucleic acid assay, as a rapid portable, ultra-sensitive, and low-cost target nucleic acid sequence assay, can be used to detect *A. citrulli* (26, 27). In this study, the extraction of watermelon acidophilus genome gDNA takes 1 h, CRISPR/LbCas12a system trans-cutting takes 90 min, then we can find the results after a UV lamp. The use of kit methods to extract genomic DNA is time-consuming, and the use of an ELISA reader to detect CRISPR/LbCas12a system trans-cutting is also for a long time. If the rapid extraction of bacterial genome gDNA, portable equipment, and CRISPR/LbCas12a non-amplification detection are combined, it can be applied to rapid detection and further research expansion. PCR is not easy to implement in portable devices, and to achieve ultra-sensitive nucleic acid detection, many scholars have also developed several alternative isothermal amplification methods in combination with Cas12 to enhance the sensitivity (28, 29), which requires additional design of specific primer preamplification signals. Currently, more researchers tend to amplification-free detection of target genes. Fozouni et al. developed a portable device technology, based on CRISPR/Cas13a to detect novel coronaviruses (30), which can be reached in 30 min by using a mobile phone under amplification-free conditions, with a sensitivity as low as 100 copies/L. The sensitivity of this device makes it difficult to realize the actual detection needs of seed samples, although it provides an opportunity for on-site detection, it is cumbersome and hard to automate the detection of samples. The amplification-free visualization method established in this study using CRISPR/Cas12a had high specificity and did not cross-react with *Vibriophilus owenii*, *Pseudomonas butyrica*, and *X. campestris*. The results of the sensitivity comparison showed that the sensitivity of CRISPR/Cas12a was 100× that of PCR and qPCR. When the CRISPR/Cas12a system was analyzed quantitatively for sensitivity, the fluorescence signal was weaker at 7 × 10^6^ copies/L than at 7 × 10^1^ copies/L and was only linear at 7 × 10^5-7^ copies/L. The fluorescence signal at 7 × 10^−2^ copies was lower than that of the negative control and failed to produce fluorescence. When 7 × 10^6^ copies/L, Cas12a, and the probe were bound when a higher concentration of the target gene was added, and the fluorescence intensity was reduced, which may be caused by the space steric effect (31), so the detection limit was as low as 7 copies/L. Although the existing literature reports the sensitivity of Cas12-based nucleic acid detection (at the attomole level), there are still some shortcomings that need to be overcome (32). On the one hand, it is necessary to select appropriate, conservative, and specific target crRNA sites, while this study only explored the “TTTN” site. Different target sites will affect the viability of crRNA, and more target sites need to be explored. On the other hand, for Cas protein itself or existing catalytic nucleic acids, the disadvantage is that it is not possible to do the test of lower sensitivity attomole without amplification, and it is still challenging to approach their attomole sensitivity (33). CRISPR/Cas12a system combined with portable devices for rapid detection of target genes without amplification conditions will become a new trend in pathogen detection research.
